# Localized ^1^H-NMR spectroscopy in patients with fibromyalgia: a controlled study of changes in cerebral glutamate/glutamine, inositol, choline, and *N*-acetylaspartate

**DOI:** 10.1186/ar3072

**Published:** 2010-07-07

**Authors:** Nicolas Fayed, Javier Garcia-Campayo, Rosa Magallón, Helena Andrés-Bergareche, Juan V Luciano, Eva Andres, Julián Beltrán

**Affiliations:** 1 Department of Radiology, Hospital Quirón, Paseo de Mariano Renovales, 50006, Zaragoza, Spain; 2 Instituto Aragonés de Ciencias de la Salud, Department of Psychiatry, Hospital Miguel Servet, Avda Isabel La Católica 1, 50009, Zaragoza, Spain; 3 REDIAPP "Red de Investigación en Actividades Preventivas y Promoción de la Salud" (RD06/0018/0017), Av. Gran Via de les Corts Catalanes, 587 Ático, 08007 Barcelona, Spain; 4 Instituto Aragonés de Ciencias de la Salud, Arrabal Health Centre, Calle Andador Aragües del Puerto 2-4, 50015, Zaragoza, Spain; 5 Sant Joan de Déu-SSM, Fundación Sant Joan de Déu, c/Dr Antoni Pujadas 4, 08830, Sant Boi de Llobregat, Barcelona, Spain; 6 CIBER Epidemiología y Salud Pública, Unidad Epidemiología Clínica, Hospital 12 de Octubre, Avda de Córdoba s/n, 28041, Madrid, Spain

## Abstract

**Introduction:**

The purpose of this study was to investigate whether single-voxel (SV) proton magnetic resonance spectroscopy (MRS), diffusion-weighted imaging (DWI), and diffusion tensor imaging (DTI) detected differences between fibromyalgia (FM) patients and healthy controls. We also searched for correlations between neuroimaging abnormalities and neuropsychological variables.

**Methods:**

Ten patients with FM and 10 gender- and age-matched control subjects were studied. A neuropsychological examination, DWI, DTI, and proton MRS were performed on the brain areas known to be associated with pain processing.

**Results:**

Compared with healthy controls, FM patients had significantly higher levels of glutamate + glutamine (Glx) (mean ± SD, 10.71 ± 0.50 arbitrary institutional units versus 9.89 ± 1.04; *P *= 0.049) and higher glutamate + glutamine/creatine (Glx/Cr) ratios (1.90 ± 0.12 versus 1.72 ± 0.23; *P *= 0.034) in the posterior gyrus. Myoinositol (Ins) levels of the right and left hippocampi were significantly lower in FM patients (4.49 ± 0.74 versus 5.17 ± 0.62; *P *= 0.008 and 4.91 ± 0.85 versus 6.09 ± 0.78; *P *= 0.004, respectively). In FM patients, decreased myoinositol/creatine (Ins/Cr) ratios were found in the left sensorimotor area (*P *= 0.05) and the left hippocampus (*P *= 0.002) and lower levels of choline (*P *= 0.019) and *N*-acetyl aspartate + *N*-acetyl aspartyl glutamate (NAA + NAG) (*P *= 0.034) in the left hippocampus. Significant correlations between depression, pain, and global function and the posterior gyrus Glx levels and Glx/Cr ratios were observed.

**Conclusions:**

Glx within the posterior gyrus could be a pathologic factor in FM. Hippocampal dysfunction may be partially responsible for the depressive symptoms of FM. Additional studies with larger samples are required to confirm these preliminary data.

## Introduction

Fibromyalgia (FM) is a prevalent and disabling disorder characterized by a history of widespread pain for at least 3 months and patient-reported tenderness in at least 11 of 18 defined tender points on digital palpation with about 4 kg per unit area of force [1]. With an estimated lifetime prevalence of approximately 2% in a community sample, FM accounts for 15% of outpatient rheumatology visits and 5% of primary care visits [2]. The prognosis for symptomatic recovery is generally poor [3]. No clear consensus exists on the treatment of choice, and FM remains relatively refractory to treatment [4].

The use of neuroimaging techniques for the study of FM has increased in recent years [5]. Some of the different neuroimaging methods used in this condition have been multiple positron-emission tomography (PET) [6], single-photon emission computed tomography (SPECT) [7], functional magnetic resonance imaging [8,9], and diffusion-tensor and volumetric imaging [10]. DTI has been effectively used for the investigation of pain. Some studies with this technique suggest that some brain structures are activated during painful conditions in healthy controls [11]. More recently, a growing body of literature suggests that glutamate (Glu), an excitatory neurotransmitter within the central nervous system, may play a role in FM pathology [12,13].

MRS provides a noninvasive method for characterizing chemical and cellular features *in vivo*. MRS can be used to measure the chemical composition of tissues, characterize certain tissue metabolic processes, and identify unanticipated chemical or metabolic relations with disease. In brain tissue, the concentrations and mobility of MRS-visible low-molecular-weight chemicals are measured as spectral peaks and can be used to detect abnormalities in brain regions that seem normal in magnetic resonance imaging (MRI) and to elucidate the pathology underlying MRI-visible abnormalities [14].

Diffusion-weighted imaging (DWI) is a sensitive tool that allows physiological alterations in water diffusion to be quantified. The apparent diffusion coefficient (ADC) is a measure of the magnitude of diffusion of the water molecules and reduced restriction, generally as the result of expansions of the extracellular space. These expansions could be produced by neural loss, glial loss, astrocytosis, or the presence of any other pathologic changes [15].

White matter composes half of the human brain and consists of bundles of myelinated axons connecting neurons in different brain regions. Gray matter is composed of neuronal cell bodies and dendrites concentrated in the outer layers of the cortex. Microstructural changes in white matter can be revealed by specialized MRI brain-imaging techniques such as diffusion tensor imaging (DTI). DWI and DTI provide evidence that subtle abnormalities exist in neuronal function in pain-processing regions of the brain, in particular, in fibromyalgia, suggesting that thalami play a significant role in the pain processing in FM [16].

DTI analyzes the fractional anisotropy (FA) of proton diffusion in tissue, which is more restricted in white matter than in gray matter. FA is a measure of the directionality of diffusion. In a healthy brain, the movement of water is expected to be anisotropic, preferentially flowing in one direction, such as along white-matter tracts, without much movement into the space outside the axon. Anisotropy increases with increased myelination, fiber diameter, and axon compaction. These data can be used to calculate the probable anatomy of white-matter fiber bundles in living brains, a process called tractography. The fiber orientation is calculated from the eigenvectors that define proton diffusion in three dimensions in each voxel. With algorithms, the principal eigenvalue vector is connected to the next voxel to trace the fiber structure and orientation in white-matter tracts [15].

Nearly all published works have focused on the diagnostic ability of a single imaging technique rather than on a comparison between several techniques. It is therefore useful to define the relative utility of these MRI techniques for distinguishing between FM and control patients. The aim of this study was to investigate whether single-voxel (SV) MRS, DWI, and DTI measurements can detect cerebral abnormalities in fibromyalgia patients and whether significant differences exist between these patients and normal controls. In addition, we searched for any possible correlations between neuroimaging abnormalities and relevant clinical variables of FM.

## Materials and methods

### Method

This was a controlled, cross-sectional study.

### Patients

The FM group was recruited from any of the primary health care centers in the city of Zaragoza, Spain. The patients were required to meet the following inclusion criteria: be 18 to 65 years old and be able to understand and read Spanish, fulfill the criteria for primary fibromyalgia according to the American College of Rheumatology [1], and have been diagnosed with fibromyalgia during the last 2 years, and have discontinued pharmacologic treatment 1 week before the study began or have modified the treatments so as not to affect brain imaging. All the patients with FM included in the study took analgesics such as tramadol (10 of 10), pregabalin (eight of 10), or paracetamol (five of 10). In addition, some of them took the antidepressant duloxetine (six of 10) and benzodiazepines (three of 10). Treatment was discontinued 1 week before the study. During that week, patients were allowed to take only occasional doses (maximum, one per day) of analgesics such as tramadol or paracetamol, to minimize the influence of medication on brain imaging. Patients were excluded if they had Axis I psychiatric disorders (depressive disorder, bipolar disorder, dementia, schizophrenia, paranoid disorder, or alcohol and/or drug-abuse disorders), were pregnant or lactating, and or if they refused to participate.

The control group was recruited from among hospital staff, with an adjustment for gender and age (± 3 years) to match the FM group by using the "head-by-head" method [17].

### Measurements

#### Clinical variables

##### Sociodemographic data

Data on the gender, age, marital status, education, and occupation of subjects were collected.

##### Pain Catastrophizing Scale (PCS)

This construct involves an exaggerated negative orientation toward noxious stimuli. The PCS is a 13-item self-reported questionnaire whose validity and reliability were previously reported [18]. We used the validated Spanish version of this questionnaire [19].

##### Hospital Anxiety Depression Scale (HADS)

This is a self-reported scale designed to screen for the presence of depression and anxiety disorders in medically ill patients. It contains 14 items rated on 4-point Likert-type scales. Two subscales assess depression and anxiety independently (HADS-Dep and HADS-Anx, respectively) [20]. Patients with 14 or more points in the complete scale (or more than 8 in either of the two subscales) were considered "probable cases" of anxiety or depression or both. We used the validated Spanish version of this questionnaire [21].

##### Pain Visual Analogue Scale (PVAS)

The PVAS was designed to allow a thorough and understandable subjective assessment of pain. A Visual Analogue Scale is usually a 10-cm horizontal line, with perpendicular lines on the edges, defined as the extreme limits of pain experience. Anchoring points at each edge are characterized by verbal expressions such as 'No pain' (accompanied by the number 0) at one edge and 'maximum pain ever experienced' (accompanied by the number 100) at the other edge. The adequate psychometric properties of PVAS were demonstrated in previous studies [22]. We used the validated Spanish version of this questionnaire.

##### Pain threshold assessed by sphygmomanometer

It has been demonstrated that the sphygmomanometer, a universally used clinical test, is helpful in the identification of patients with FM [20]. It has been recommended that the blood pressure cuff be inflated at an approximate rate of 10 mm Hg per second up to 180 mm Hg or to the point at which pain is elicited. Healthy persons usually experience pain when the blood pressure cuff is inflated to 160 mm Hg or more; patients with FM, though, usually experience pain at 100 to 110 mm Hg, or even with less pressure [23].

##### Mini-Mental State Exam (MMSE)

This is a fully structured scale that consists of 30 points grouped into seven categories: orientation to place, orientation to time, registration, attention and concentration, recall, language, and visual construction. The psychometric properties of the test were previously described. In nongeriatric populations (younger than 65 years), such as the sample in our study, the threshold that suggests a 'probable case' of cognitive disorder is less than 27 points [24]. We used the validated Spanish version of this questionnaire [25].

##### Fibromyalgia Impact Questionnaire (FIQ)

The FIQ is a 10-item self-reported questionnaire developed to measure the health status of FM patients [26]. The first item focuses on the patient's ability to carry out muscular activities. In the next two items, patients are asked to circle the number of days in the past week on which they felt good and how often they missed work. Finally, the last seven questions (job ability, pain, fatigue, morning tiredness, stiffness, anxiety, and depression) are measured with a visual analogue scale (VAS). We used the validated Spanish version of this questionnaire [27].

#### Neuroimaging techniques

##### Magnetic resonance imaging (MRI)

Data were acquired by using a 1.5-T Sigma HD clinical scanner (GE Healthcare Diagnostic Imaging, Milwaukee, WI, USA). All images were acquired by using an eight-channel phased-array head coil (NVHEAD A). For each subject, a three-dimensional high-resolution (1-mm isotropic voxels) structural magnetic resonance image was acquired by using a T_1_-weighted volumetric spoiled gradient-recall echo sequence (repetition time (RT) = 9.1 ms; echo time (ET) = 1.7 ms; inversion time (IT) = 450 ms, 20-degree flip angle, number of excitations = 1; matrix size = 256 × 160; slice thickness = 1.5 mm; yielding 140 coronal slices with an in-plane resolution of 1 × 1 mm).

##### Functional magnetic resonance imaging (*f*MRI)

*f*MRI acquisition consisted of a single-shot gradient-echo echo-planar imaging (EPI) sequence, which was used to acquire repeatedly a series of T_2_-weighted images (FOV = 24 cm; TE/TR/flip = 60/3,000 msec/90 degrees; slice thickness/gap = 5/0 mm; matrix size = 64 × 64) at 25 slice locations encompassing the motor cortex region. In total, 64 images were collected at each slice location, which correspond to a series of four dummy acquisitions followed by 6 times periods of 10 volumes each. Motor cortex stimulation was alternated with rest periods, with each period lasting 30 sec. During the task period, the subjects were asked to perform a self-paced finger-thumb opposition of the contralateral hand. These images were subsequently processed off-line by using the commercial clinical software FuncTool 3.1.23 (General Electric Medical System, Milwaukee, WI). Preprocessing of the functional MR data included motion correction, slice-time correction, and temporal drifting correction. The Student *t *test was used with a confident level of 0.001 to identify the statistically significant pixels. The co-registration of the *t-*test maps with the anatomic image was performed to obtain the anatomic localization of the functional foci.

##### Magnetic resonance spectroscopy (MRS)

**Figure 1:**
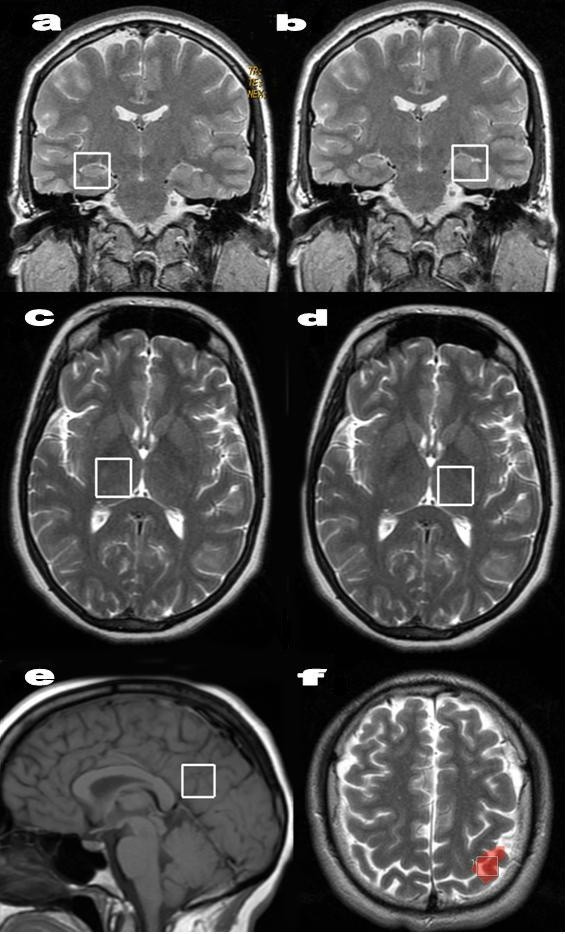
**Voxel placement in the different brain regions**. The hippocampus **(a, b)**, thalamus **(c, d)**, posterior gyrus **(e)**, and left sensorimotor **(f) **(red) after functional magnetic resonance imaging during finger-tapping tasks.

An axial T_2_-weighted image was used to locate volumes of interest (VOIs) (2 × 2 × 2 cm) in the left primary sensorimotor area and both halves of the thalamus. A coronal T_2_-weighted image in the plane that goes through inner auditive conducts and brain peduncle was used to locate volumes in both hippocampi, and a midsagittal T_1_-weighted image was used to locate a voxel on the posterior gyrus (Figure 1). A close negative correlation between global measurement-scale scores and NAA levels in the gray matter of the posterior cingulate has been shown in cognitive impairment [28-30], and cognitive dysfunction is a key symptom in FM.

**Figure 2:**
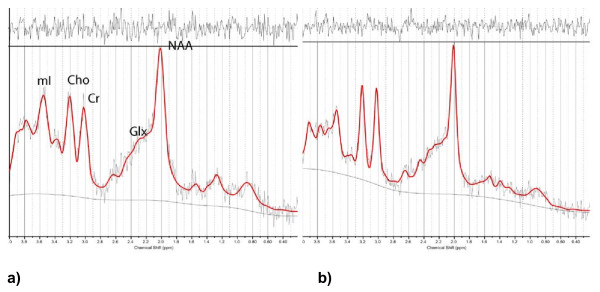
**Left hippocampus spectrum**. A control patient **(a) **and a patient with fibromyalgia (FM) **(b)**. For the patient with FM, a decrease in the myoinositol peak amplitude and in the relation to creatine was noted. The black line indicates the averaged spectrum; the red line indicates the LCModel fit. The residuals, calculated as a subtraction of the fit from the average spectrum, are plotted at the top.

^1^H-MRS was carried out by means of a short echo-time (ET) of 35 msec and a repetition time (RT) of 2,000 msec and 128 accumulations by using a single-voxel stimulated-echo acquisition-mode localization sequence with a spin-echo technique that uses selective excitation with gradient spoiling for water suppression. The mode of spectral acquisition was probe-p (PRESS technique). Chemical concentrations can now be automatically extracted from MR spectra by using sophisticated and well-documented time-domain and spectral frequency-fitting software packages such as LCModel (Stephen Provencher, Oakville, Ontario, Canada), a user-independent fitting routine based on a library of model spectra of all individual metabolites [31]. Concentration values are expressed as arbitrary institutional units and are not corrected for contributions by CSF and a small reduction in the numeric values by residual T_1 _and T_2 _relaxation effects. The data evaluation comprised a correction of the spectroscopic time-domain data for residual eddy-current effects. Quantifiable chemicals by MRS included the following: *N*-acetylaspartate (NAA; most often measured as the total of NAA + *N-*acetylaspartyl glutamate [Glu]), 2.02 ppm; glutamine and glutamate (Glx), 2.1 to 2.55 ppm; total creatine (Cr; composed of creatine and phosphocreatine), 3.03 ppm; choline-containing compounds (Cho), 3.23 ppm; and myoinositol (mI), 3.56 ppm (Figure 2). We also obtained the ratios of the peak amplitude of the metabolites relative to creatine. The areas of exploration were chosen on the basis of brain structures that are activated during painful conditions in healthy controls, the somatosensory cortices and the thalamus, and the regions in the previously mentioned reports implicated in cognitive impairment, the hippocampus and posterior gyrus [32]. Before starting this study, we studied the test-retest reliability of metabolite measurements in every area in a sample of patients with other pathologies, with two consecutive studies, without removing the patient from the scanner. According to the resulting α coefficients, we must assume a mean random variation in the posterior gyrus of around 8% for mI/Cr and of around 10% for NAA/Cr, Cho/Cr, and glutamate [33].

##### Diffusion tensor imaging (DTI)

These images were obtained by using a single-shot, spin-echo EPI technique with a b-value of 1,000 s/mm^2 ^for each of 25 diffusion-encoding directions, ET = 94.5 ms, TR = 8,000 ms, matrix = 128 × 128, field of view, 24 cm, 3-mm slice thickness with no gaps, 25 slices, and a scan time of 3.44 min. The diffusion MR data were analyzed by using the diffusion tensor model. After a mathematical diagonalization process, the eigenvectors and eigenvalues describing the tensor ellipsoid were determined. Subsequently, two standard diffusion indices were derived: the apparent diffusion coefficient (ADC) and the fractional anisotropy (FA) [13]. The ADC and FA maps were calculated off-line with the Functool software 3.1.23 in the Advantage Workstation 4.3 (General Electric Medical Systems, Milwaukee, WI) by the following procedure. Initially, images were preprocessed to remove image-to-image misregistration that arises from directional eddy currents during echo-planar readout. Directional diffusion-weighted images (DWIs) were spatially registered to the b ≅ 0 image, which was set to remove image shear, compression, and shift by an affine transform. ADC is considered quantitative with normal brain values of ADC ≅ 0.7 × 10^-3 ^mm^2^/sec, and FA is a dimensionless value between zero (isotropic) and close to 1 (highly anisotropic environments).

**Figure 3:**
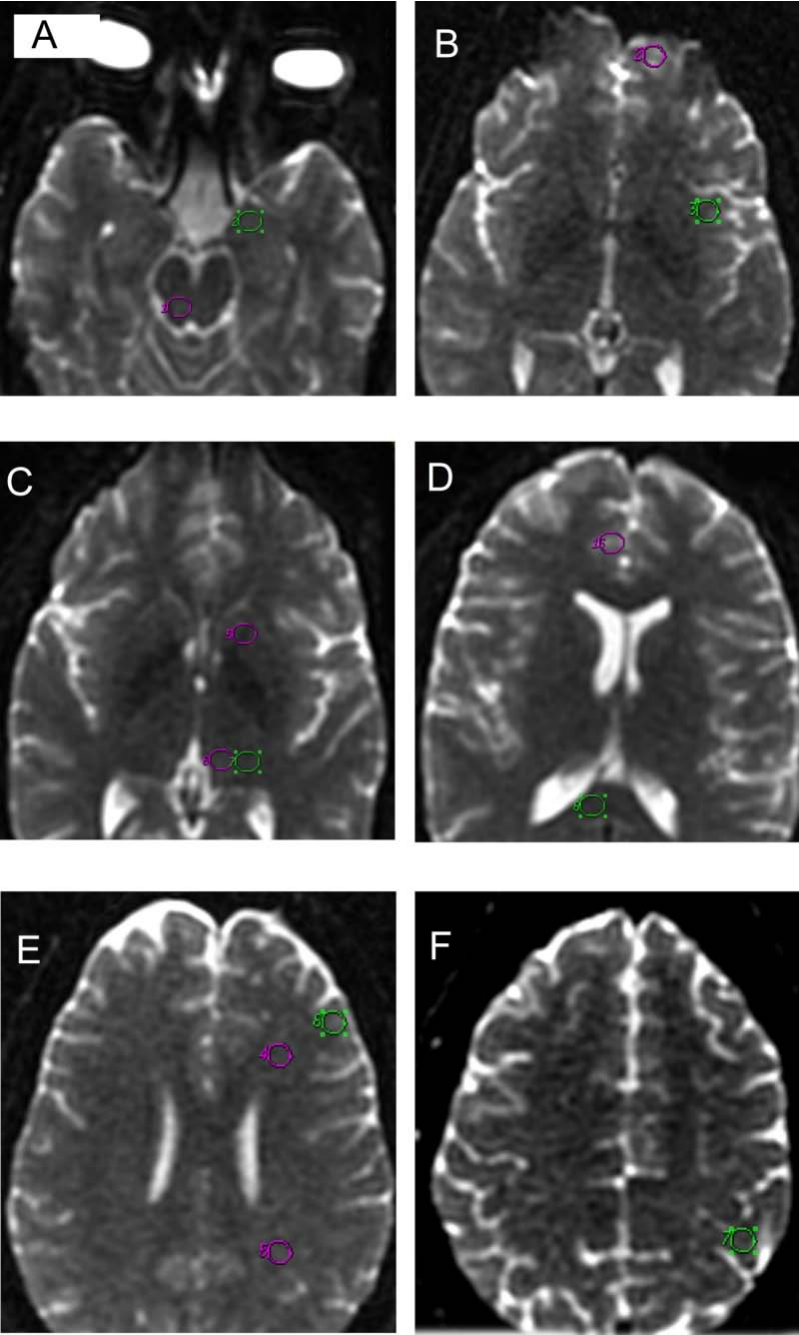
**Axial images showing the different locations of the equal-sized regions of interest (ROIs)**. The ROI placements for the periaqueductal gray and amygdale **(a)**, the orbitofrontal cortex and insular cortex **(b)**, the internal capsule and ventral and dorsolateral thalamus **(c)**, the gyrus cortex and corpus callosum **(d)**, the frontal white matter, parietal white matter, and dorsolateral prefrontal cortex **(e)**, and the sensorimotor area **(f)**.

Standardized 50-mm^3 ^circular regions of interest (ROIs) were placed at the following areas, most of them known to be associated with pain processing: the lemniscus medial, amygdala, periaqueductal gray, insular cortex, orbitofrontal cortex, internal capsule, middle thalamus, dorsolateral thalamus, corpus callosum, dorsolateral prefrontal cortex, gyrus cortex, parietal and frontal white matter, and somatosensory cortex (Figure 3). The mean ADC and FA in the different regions were compared between the two groups.

The total scanning time for six MRS acquisitions (30:20), a T_1_-weighted volumetric spoiled gradient-recall echo sequence (5:39), T_1_- (1:39) and T_2_-weighted scans (1:11), an *f*MRI run (3:12), and DWI (3:44) was 44:65.

### Blood tests

Venous blood was drawn between 9:00 am and 11:30 am. When the blood was extracted, it was placed into two containers for separation:

Container (a): A container with only serum. This was centrifuged and coagulated. In this sample, lactate dehydrogenase (LDH) and serotonin were studied.

Container (b): A container with blood. This was centrifuged, and an anticoagulant (EDTA tripotasic) was added. This was frozen at -30°C Celsius until use. In this sample, ammonia was studied.

Ammonia and LDH levels were measured with molecular absorption spectrometry, and serotonin was measured with chromatography. The results were expressed as individual patient levels and reference values, by using micromoles per liter and micrograms per deciliter (ammonia), microKat per liter and Units per liter (LDH) and nanomoles per liter and nanograms per milliliter (serotonin).

### Statistical methods

To describe the quantitative variables, the means and standard deviations were calculated. A nonparametric Mann-Whitney *U *test was used to analyze possible differences in brain metabolite levels between patients with fibromyalgia and healthy controls. In addition, we used the nonparametric Spearman's rho correlation in the FM group to study the relation between brain metabolites for which the levels were significantly different in FM patients compared with those in healthy controls, and the clinical variables were studied. To determine the statistical significance in psychological and blood tests between the controls and patients with FM, a Mann-Whitney *U *test was used. Statistical analyses were carried out by using SPSS 15.0, and *p *values lower than 0.05 were considered statistically significant for all analyses.

### Ethical aspects

Informed consent was obtained from the participants before their inclusion in the study. The study protocol was approved by the ethical review board of the regional health authority.

## Results

### Sociodemographic and psychological variables and pain measurements

Ten patients (eight women and two men) with FM and 10 gender- and sex-matched control subjects were studied. The mean age of FM patients was 40.0 (SD = 6.2) years, and the mean age in the control group was 37.8 (SD = 8.7) years. As expected, no significant differences were found in these two variables because of a matching process. Neither group had any psychiatric disorder, as was confirmed by the SPPI psychiatric interview. In the control group, rating scores in the psychopathology questionnaires were within the normal ranges.

In the FM group, the mean duration of the disorder was 1.6 (SD = 0.3) years. The psychological profile showed the usual psychological characteristics of FM patients: high scores in anxiety (mean, 6.2; SD, 2.8) and depression (mean, 5.9; SD, 3.8) assessed with HADS; high scores on the pain-catastrophizing scale (mean, 27.0; SD, 9.9) and in pain assessed with PVAS (mean, 63.0; SD, 17.0); and a low pain threshold in the sphygmomanometer test (mean, 107.0; SD, 25.8). The MMSE scores suggested a certain cognitive dysfunction in FM (mean, 30.6; SD, 3.3), but it was not as severe as that found in patients with dementia.

### Blood test

The blood concentration of 5-hydroxytryptamine (serotonin) decreased significantly in the FM group, with a range from 21 to 229 n*M *(mean, 77.3). Two patients with FM showed an increased level of lactate dehydrogenase (mean, 85.2 nKat/L). Table 1 summarises psychological and blood-test variables in the controls and in the patients with FM.

**Table 1:** Psychological and blood-test variables in controls and patients with FM

Variables	Healthy group (mean ± SD)	FM group (mean ± SD)	*P *Value
Psychological variables			

PVAS	16.50 ± 5.79	63.0 ± 17.02	< 0.001
Sphigmo	170.50 ± 7.24	107.0 ± 25.84	< 0.001
MMSE	34.90 ± 0.31	30.60 ± 3.37	< 0.001
Catastroph	4.90 ± 2.02	27.0 ± 9.98	< 0.001
FIQ	17.50 ± 4.85	70.20 ± 10.50	< 0.001
HADS-Anx	0.70 ± 0.67	7.40 ± 0.69	< 0.001
HADS-Dep	0.60 ± 0.51	6.90 ± 0.87	< 0.001

Blood-test variables			

Serotonin	273.50 ± 10.42	297.56 ± 249.70	0.449
GDH	47.10 ± 2.23	48.33 ± 23.51	0.568
Ammonium	17.40 ± 1.34	28.32 ± 9.26	< 0.001

All calculations were performed by using the Mann-Whitney *U *test.

Catastroph, Pain catastrophization scale; FIQ, Fibromyalgia Impact Questionnaire; GDH, glutamate dehydrogenase; HADS-Anx, Hospital Anxiety Depression Scale, subscale anxiety; HADS-Dep, Hospital Anxiety Depression Scale, subscale depression; MMSE, Mini Mental State Examination; PVAS, Pain Visual Analogue Scale; Sphigmo, pain assessed by sphygmomanometer.

### Neuroimaging variables

#### MRI

The conventional MR images were normal with respect to the brain parenchyma in all subjects.

#### DWI and DTI

With regard to diffusion-weighted images and diffusion-tensor imaging, we did not detect significant differences in any of the areas.

#### MRS

FM patients showed increased combined glutamine and glutamate (that is, Glx) and decreased myoinositol levels. Table 2 shows that individuals with FM displayed elevated glutamate+glutamine (Glx) and an elevated Glx/Cr ratio in the posterior gyrus (*P *= 0.049 and *P *= 0.034, respectively) compared with healthy controls. Myoinositol (Ins) levels in the right and left hippocampi were significantly lower in FM patients compared with controls (*P *= 0.008 and *P *= 0.004, respectively). FM patients also showed lower myoinositol/creatine (Ins/Cr) ratios in the left sensorimotor area (*P *< 0.05) and the left hippocampus (*P *= 0.002) compared with controls. An example of a spectrum is given in Figure 2. Another statistically significant difference was observed for choline (*P *= 0.019) and NAA+NAG (*P *= 0.034) in the left hippocampus, with levels being lower in the patient group compared with controls.

**Table 2:** Comparison of absolute and relative Cr metabolite levels between FM patients and healthy controls

Metabolite location	Healthy group	FM group	*P *value
Posterior cingulate gyrus			

Glx	9.89 ± 1.04	10.71 ± 0.50	0.049
Glx/Cr	1.72 ± 0.23	1.90 ± 0.12	0.034

Left hippocampus			

Ins	6.09 ± 0.78	4.91 ± 0.85	0.004
Ins/Cr	1.22 ± 0.07	1.09 ± 0.08	0.002
Cho	1.72 ± 0.20	1.47 ± 0.22	0.019
NAA+NAAG	7.05 ± 0.64	6.27 ± 0.89	0.034

Right hippocampus			

Ins	5.17 ± 0.62	4.49 ± 0.74	0.008
Sensorimotor cortex			
Ins/Cr	0.84 ± 0.07	0.76 ± 0.09	0.05

Values are expressed as the mean ± SD (in arbitrary institutional units).

Cho, choline; Cr, creatine; FM, fibromyalgia; Glx, glutamate+glutamine; Glx/Cr, glutamate+glutamine/creatine ratio; Ins, myoinositol; Ins/Cr, myoinositol/creatine ratio; NAA+NAAG, *N*-acetylaspartate + *N*-acetylaspartyl glutamate.

**Figure 4:**
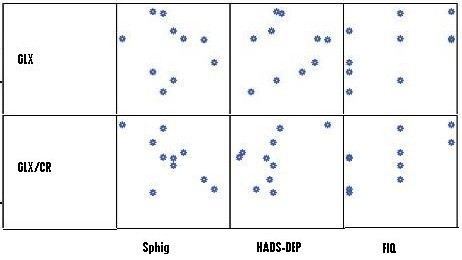
**Scatterplot of the correlations between brain metabolites and psychological variables in patients with FM**. FIQ, Fibromyalgia Impact Questionnaire; Glx, glutamate+glutamine; Glx-Cr, glutamate+glutamine/creatine; HADS-dep: Hospital Anxiety Depression Scale (depression subscale); Sphig, pain assessed with sphygmomanometer (the pressure that is needed to evoke pain). Blue circles represent patients with FM.

Regarding Glx and Glx/Cr in the posterior gyrus and psychological variables, significant correlations between depression, pain measured with a sphygmomanometer, and global function measured with FIQ and the posterior gyrus Glx and Glx/Cr levels were observed (Table 3). The scatterplot of the correlations between brain metabolites and psychological variables that are significantly different between patients with FM and controls are shown in Figure 4. These data suggest that, regardless of whether an individual is an FM patient or a healthy control, individuals with higher levels of Glx and Glx/Cr also have enhanced sensitivity to induced pressure pain.

**Table 3:** Correlations between brain metabolites that show significant differences between both groups and psychological variables

Metabolite	HADS-anx	HADS-dep	PVAS	Sphigm	MMSE	Catastroph	FIQ
Posterior cingulate gyrus Glx							

(Coefficient)	0.43	0.44	0.37	-0.45	0.39	0.40	0.45
(*P *value)	0.057	0.047^a^	0.107	-0.047^a^	0.089	0.081	0.043^a^

Posterior cingulate gyrus Glx/Cr ratio							

(Coefficient)	0.42	0.47	0.39	-0.50	0.36	0.40	0.55
(*P *value)	0.062	0.034^a^	0.08	-0.024^a^	0.117	0.077	0.011^a^

^a^*P *< 0.05.

Catastroph, Pain catastrophization scale; FIQ, Fibromyalgia Impact Questionnaire; Glx, glutamate+glutamine; Glx/Cr, glutamate+glutamine/creatine ratio; HADS-Anx, Hospital Anxiety Depression Scale, subscale anxiety; HADS-Dep, Hospital Anxiety Depression Scale, subscale depression; MMSE, Mini Mental State Examination; PVAS, Pain Visual Analogue Scale; Sphigm, pain threshold assessed by sphygmomanometer (the pressure that is needed to evoke pain).

## Discussion

In this pilot study, we compared three different magnetic resonance imaging (MRI) examination methods for the diagnosis of fibromyalgia: magnetic resonance spectroscopy, diffusion-weighted imaging, and diffusion-tensor imaging. To our knowledge, this is the first controlled study to compare these different neuroimaging examination methods directly in patients with fibromyalgia. The main limitations of this study are the small sample size (*n *= 10), as is usual in pilot studies, and the fact that this exploratory study did not attempt to control for multiple comparisons. Finally, the interpretation of significant differences in the metabolite concentrations should be cautious because spectroscopic voxels contain varying mixtures of gray matter, white matter, and CSF.

### Performance of different neuroimaging techniques for the diagnosis of FM

Previous studies with diffusion-tensor imaging in FM patients showed alterations in the right thalamus and significantly lower fractionated anisotropy compared with controls. A negative correlation was seen between the FA values in the right thalamus and clinical pain in the FM group [16]. Other authors confirmed that DTI in the brains of patients with FM appears to be more sensitive than volumetric imaging of voxel-based morphometry (VBM), and increased pain-intensity scores were correlated with changes in DTI measurements in the right superior frontal gyrus. Increased fatigue was correlated with changes in the left superior frontal and left anterior gyri, and self-perceived physical impairment was correlated with changes in the left postcentral gyrus [10].

In our study, the DWI and DTI were not sensitive enough to detect changes in fibromyalgia patients. This may be explained because the intraparenchymal injection of glutamate or other excitatory amino acids predominantly affects neuronal cell bodies and dendrites, whereas axons and terminal boutons, originating from cell bodies outside of the affected region, remain largely intact [34]. In addition, only one average was acquired for DWI; this would be expected to have a rather low signal-to-noise ratio, and therefore, negative findings have to be considered with caution.

### Correlations between brain metabolites and pain and psychological variables

In our study, a significant correlation was noted in the posterior gyrus between Glx levels and Glx/Cr ratios with depression (higher levels of glutamate correlated with higher depression scoring), pain measured with a sphygmomanometer (high levels of glutamate correlated with lower sphygmomanometer pressure, which means more proneness to experiencing pain), and global function assessed with FIQ (higher levels of glutamate levels correlated with worse global function). Because astrocytes participate in the uptake, metabolism, and recycling of glutamate, we hypothesize that an astrocytic deficit may account for the alterations in glutamate/GABA neurotransmission in depression. Factors such as stress, excess glucocorticoids, altered gene expression of neurotrophic factors and glial transporters, and changes in extracellular levels of neurotransmitters released by neurons may modify glial cell numbers and affect the neurophysiology of depression [35]. Other studies found that the absolute concentrations of glutamate+glutamine (Glx), glutamate (Glu), and creatine+phosphocreatine (Cr) were significantly higher in adult bipolar patients in all mood states compared with healthy controls [36].

Glutamate has been implicated as an important mediator in the neurotransmission, potentiation, and negative effects associated with pain, and it has been related to chronic pain sensitization [37]. Our data suggest that Glu plays a role in this augmented pain processing in those individuals who have elevated Glu levels. Because higher Glu levels were associated with lower pain thresholds, it is likely that Glu in the posterior cingulate is related to pain processing. The elevated levels of Glu in the FM group could raise the set point of baseline neural activity in this region, which could result in augmented responses to painful stimuli [38].

FM patients may have more Glu within their synaptic vesicles, higher numbers or densities of glutamatergic synapses, or even less uptake of Glu from the synaptic cleft. Any of these changes would be consistent with the hypothesis that augmentation of pain and sensory processing is found in FM. FM may represent a condition in which glutamatergic "hyperactivity" occurs within brain regions devoted to processing and modulating pain. The results suggested increased glutamatergic metabolism in exacerbated chronic illness or as a medication effect.

An alternative hypothesis is that the increase of glutamine in astrocytes could precipitate a cascade of metabolic events that will ultimately result in neuronal dysfunction [39]. One of the consequences of the increased glutamine levels in astrocytes is cellular swelling, which has been demonstrated in preparations of astrocytes and in experimental models in rats [40]. The findings of ^1^H-MR spectroscopy in humans are in accordance with the development of astrocyte swelling. The intraparenchymal injection of glutamate predominantly affects neuronal cell bodies and dendrites [34]. Because astrocytes participate in the uptake, metabolism, and recycling of glutamate, we hypothesize that an astrocytic deficit may account for the alterations in glutamate/GABA neurotransmission in FM.

### Differences in brain metabolites between patients with FM and controls

#### Glutamate+Glutamine

One of the principal findings in our study was the increase in the combined glutamate+glutamine (Glx) levels and glutamate+glutamine/creatine ratio (Glx/Cr) in the posterior gyrus (Figure 3) in individuals with FM compared with controls. Because higher Glx levels were associated with lower pain thresholds, this suggests that Glx in the posterior gyrus is related to pain processing. The elevated levels of Glx in the FM group could raise the set point of baseline neural activity in this region, which could result in augmented responses to painful stimuli. It has been demonstrated that painful stimuli elicit a dynamic increase in glutamate in the anterior cortex [40]. Increases in glutamine levels also were seen, and these levels correlated strongly with the subjective level of pain experienced by participants [41]. Other studies have shown an increase in glutamate and Glx in the right posterior insula, which is associated with lower pressure-pain thresholds [11,12].

Elevated glutamine and glutamate inside the astrocytes leads to increased cellular osmolarity in the brain. Within 30 minutes of glutamate administration, electron microscopy reveals massive acute swelling of neuronal cell bodies and dendrites. Consequently, water shifts from the extracellular fluid space to the intracellular fluid space, resulting in edema of the astrocytes [42]. Relevant clinical manifestations are thought to be secondary to this edema [39]. To compensate for the increased cellular osmolarity, myoinositol shifts to the extracellular space, leading to reduction in its concentration inside the astrocyte. In consequence, glutamate+glutamine (Glx) is an excitatory amino acid, and its increase indicates that the metabolic function of patients with fibromyalgia differs from that of controls. The elevation of Gln levels may result in permanent cerebral damage.

#### Choline and NAA+NAAG

Our study confirms the reduction in choline and NAA+NAAG in the left hippocampus (*P *= 0.019 and *P *= 0.034, respectively). In all the cases, brain metabolites were lower in the patient group compared with the controls. In this study, the mean choline levels obtained in the left hippocampus were 1.47 ± 0.22 in fibromyalgia patients compared with 1.72 ± 0.20 in controls.

Choline (Cho) participates in phospholipid metabolism and osmotic regulation in glial cells. Increases in Cho resonance probably reflect increased membrane synthesis or myelin destruction, whereas decreases in Cho are associated with osmolar changes in the brain or liver diseases [43]. Disturbances of Cho and myoinositol have been interpreted as a compensatory response to the increase in intracellular osmolarity caused by the accumulation of glutamine in astrocytes [42]. The reduction in the intracellular choline (Cho) levels is also a likely mechanism to compensate for hyperosmolarity [36]. Low choline levels have been observed in hepatic encephalopathy [44] in stroke and HIV patients. Cho/Cr variability in the right dorsolateral prefrontal cortex (DLPFC) was significantly different between the FM and control groups.

The evoked-pain threshold correlated significantly with the NAA/Cho ratios in the left insula and left basal ganglia [45]. Our findings are consistent with a recent H-MRS study, which showed decreased NAA levels within the hippocampus of individuals with FM [46]. In this study, the mean NAA+NAAG levels obtained in the left hippocampus were 6.27 ± 0.89 in fibromyalgia patients compared with 7.05 ± 0.64 in the controls. In another study, a reduction in the absolute concentration of NAA of the right and left hippocampi was reported in a sample of 15 patients with fibromyalgia [47].

The lower hippocampal NAA levels suggest neuronal or axonal metabolic dysfunction, or some combination of these processes. However, neuronal loss within the hippocampus was not studied in our series, and this should be assessed in further studies to gauge atrophic changes within the hippocampus. Some studies found that the persistence of elevated Ca^2+ ^levels in hippocampal neurons exposed to glutamate correlated with the extent of neuronal death [48] and that a large increase in Ca^2+ ^in cultured hippocampal neurons after glutamate application predicted cell death [49]. We suggest that hippocampal dysfunction may be in part responsible for some of the phenomena associated with FM. Blocking *N*-methyl-D-aspartate (NMDA) receptors in the hippocampal formation reduces nociceptive behaviors; this in turn supports the hypothesis that the hippocampal formation is involved in the pain-related neural processing and expression of pain-related behaviors [50].

#### Myoinositol

Finally, our study confirmed the reduction in myoinositol levels in both hippocampi and the reduction in myoinositol/Cr ratios in the left hippocampus and sensorimotor area. In all the cases, brain metabolites were lower in the patient group compared with the controls. Changes in these brain metabolites were previously described in patients with bipolar disorder [51]. Research suggests that lithium functions primarily by decreasing myoinositol concentrations in bipolar patients [52]. In addition, patients with clinical depression generally have decreased levels of inositol in their cerebrospinal fluid [53]. A frequent association of FM with affective disorders has been described [54].

## Conclusions

According to our results, the only brain metabolite that showed a significant correlation with pain and psychological variables was glutamate + glutamine (Glx), which correlates with depression, pain, and global function. Overall, we found that Glx within the posterior gyrus was a potential pathologic factor in FM, and we speculate that myoinositol might, through its conversion to glucuronic acid, be consumed in the protective detoxification reactions of the brain. We suggest that hippocampal dysfunction may be in part responsible for some of the phenomena associated with depression in FM patients. New studies with larger samples are necessary to confirm these preliminary data. Our findings may indicate ways to find new therapeutic strategies for the treatment of patients with this puzzling syndrome.

## Abbreviations

ADC: apparent diffusion coefficient; Catastroph: pain catastrophization scale; Cho: choline; DTI: diffusion tensor imaging; DWI: diffusion-weighted imaging; FA: fractional anisotropy; FIQ: Fibromyalgia Impact Questionnaire; FM: fibromyalgia; GDH: glutamate dehydrogenase; Glu: glutamate; Glx: glutamate+glutamine; Glx/Cr: glutamate+glutamine/creatine; Ins: myoinositol; HADS-Anx: Hospital Anxiety Depression Scale: subscale anxiety; HADS-Dep: Hospital Anxiety Depression Scale: subscale depression; Ins/Cr: myoinositol/creatine; MMSE: Mini Mental State Examination; (MR)I: (magnetic resonance) imaging; MRS: magnetic resonance spectroscopy; NAA: *N*-acetylaspartate; PET: positron emission tomography; PVAS: Pain Visual Analogue Scale; ROI: region of interest; SPECT: single-photon emission computed tomography; Sphigmo: pain assessed with sphygmomanometer; SV: single-voxel; VOI: volume of interest.

## Competing interests

The authors declare that they have no competing interests.

## Authors' contributions

NF and JGC are the principal researchers and developed the original idea for the study. The study design was further developed by RM, HAB, and JVL. NF and JB carried out the neuroimage techniques. JGC, HAB, and JVL administered the psychological tests. EA developed the statistical methods. All authors read and corrected draft versions and approved the final version.
